# Drivers of alien species composition in bird markets across the world

**DOI:** 10.1002/ece3.8397

**Published:** 2021-12-24

**Authors:** Shan Su, Miquel Vall‐llosera, Phillip Cassey, Tim M. Blackburn, Martina Carrete, José L. Tella

**Affiliations:** ^1^ The Wildlife Conservation Research Unit Department of Zoology University of Oxford Oxford UK; ^2^ Research Department of Genetics, Evolution and Environment University College London London UK; ^3^ School of Biological Sciences and the Environment Institute University of Adelaide Adelaide SA Australia; ^4^ Institute of Zoology Zoological Society of London London UK; ^5^ Department of Conservation Biology Estación Biológica de Donana (CSIC) Sevilla Spain

**Keywords:** biological invasions, exotic bird trade, international pet trade, invasive species, non‐native species, non‐randomness, pet bird trade, wildlife trade

## Abstract

The global pet trade is a major pathway for the introduction of invasive alien species. The composition of species selected for transport is driven by market demands, which may be influenced by a combination of both historical and cultural factors. We compared Eastern (Taiwan) and Western (Australia and the Iberian Peninsula) bird markets to explore factors associated with the species composition and geographic origin of the birds for sale. We used a bespoke randomization test to compare species composition, geographic origins, and species overlap at different taxonomic levels among bird markets across countries. Alien species identified in the study accounted for more than 10% of the world's bird species. Parrots and songbirds were the most common alien bird taxa traded across all markets. In both Iberian and Australian markets, there was a strong bias toward parrots, waxbills, gamebirds, and finches. In Taiwan, species traded more than expected were parrots, waxbills, starlings, and leafbirds. Neotropical species were the most traded group in the three markets. Afrotropical species were also traded more than expected in Iberian and Australian markets, while the Taiwanese traded more alien species from neighboring Asian regions. The bird trade focuses on the same few bird groups worldwide. The composition and origin of species preferred in the Western markets may be influenced by colonial histories, cultural similarity, and strict regulations on wildlife importation, while species preferences in Eastern markets are strongly influenced by regional culture and proximity. Propagule pressure is a dominant factor influencing the success of biological invasions; it is important to recognize differences in the composition of bird markets among regions because they can translate into different invasion risks, among other factors.

## Graphical abstract

We compared Eastern (Taiwan) and Western (Australia and the Iberian Peninsula) bird markets to explore how these factors and the important role of globalization in the homogenizing markets are associated with the species composition and geographic origin of the birds for sale.

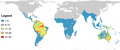

## 1 INTRODUCTION

The global pet trade is a primary pathway for the transportation of animal species outside their native ranges (Bush et al., 2014; Carrete & Tella, 2008; Edelaar et al., 2015; Lockwood et al., 2019; Seebens et al., 2017; Westphal et al., 2008). Indeed, the volume of imports to a country is positively associated with the number of invasive alien species reported (Cardador et al., 2019; Romagosa et al., 2009; Westphal et al., 2008). These species can be intentionally released or accidentally allowed to escape into the wild (Carrete & Tella, 2008, 2015; Lee & Shieh, 2005), and may subsequently establish alien populations (Abellán et al., 2016, 2017; Cardador et al., 2016), or even become invasive pests (Blackburn et al., 2009). The invasion process only commences if species are transported into a new region (Blackburn et al., 2011); therefore, knowing which species are traded provides key information about which ones might subsequently become invasive (Blackburn & Duncan, 2001). To inform measures to prevent further invasions, it is crucial to understand what shapes the species composition of pet markets.

Birds are a large component of the global pet trade (Bush et al., 2014), and traded species are not a random selection of all extant species (Abellán et al., 2016; Blackburn & Cassey, 2007; Jeschke & Strayer, 2006; Su et al., 2014). The selection of the species traded is driven largely by human demands, but these demands may differ between countries due to their cultural, historical, and geographical characteristics (Ribeiro et al., 2019). For example, in Asian countries, such as Taiwan, birds are traded for widespread traditional practices including bird song competitions, bird‐keeping, and prayer animal release (Jepson & Ladle, 2005; Nash, 1993; Severinghaus & Chi, 1999). However, in European and ex‐European colonies such as the United States, South Africa, Australia, or New Zealand, bird trade has historically been driven by the use of species for hunting, food, ornamentation, biological control (Abellán et al., 2016; Blackburn et al., 2009), and more recently by the pet trade (Abellán et al., 2016; Carrete & Tella, 2008; Dyer et al., 2017; Westphal et al., 2008). These differences in cultural and historical backgrounds suggest that the composition and origins of species in the bird trade may differ between geographic regions (Cassey et al., 2015; Edmunds et al., 2011; Jepson & Ladle, 2005; Severinghaus & Chi, 1999).

Here, we investigated the bird trade by comparing the composition and origins of alien species in the bird markets among three regions: Iberia (the markets of Spain and Portugal are combined, due to the highly shared cultural histories and affinities between both countries), Australia, and Taiwan. We chose these markets because they are likely to represent different pet demands in terms of cultural, historical, and geographical characteristics and because trends in their bird trade markets have been extensively described previously (Abellán et al., 2016; Alacs & Georges, 2008; Duncan et al., 2001; Severinghaus & Chi, 1999; Shieh et al., 2006; Su et al., 2014, 2015). The Iberian market is representative of the bird trade in Western Europe since thousands of birds from several species transported to Europe were subsequently moved among countries such as Portugal, Spain, the Netherlands, Belgium, France, Germany, the Czech Republic, and Italy (J. L. Tella, unpubl. data). The Taiwanese market is an excellent example of the Eastern bird trade culture, where prayer animal release, song competitions, and bird‐keeping are popular, as in many Asian countries, including China, Cambodia, Singapore, Thailand, or Vietnam (Eaton et al., 2017; Edmunds et al., 2011; Gilbert et al., 2012; Heinrich et al., 2020; Nash, 1993; Xu & Dong, 2007). The Australian market represents a trade that is likely to be driven by a largely Western cultural influence, but in a very different geographical context than the Iberian market.

Some of the datasets used in this study have previously been analyzed to understand the invasion risks associated with transporting alien species into different regions, as well as their characteristics. Here, we combine them for the first time, to understand what are the likely drivers determining the species composition of these markets, and how much influence can be assigned to cultural, historical, and geographic factors. If cultural and historical factors were the most important determinants of alien species composition in the bird markets, we would expect that the origin and composition of alien species for sale in Iberia and Australia would have high similarities, as both regions share Western cultural and historical influences (Turner, 2003), and biological exchanges during their colonial periods may have had a lasting influence on the composition of the traded alien species in the studied regions. In particular, we expected alien species traded in Iberia to be more likely to originate from South America and Africa due to their history of colonization (Merriman, 1962; Russell‐Wood & Russell‐Wood, 1998). In addition, the composition and origin of species in the Australian market should be more likely to be derived from ex‐British colonies, given the influences of the United Kingdom during the Victorian era. In Taiwan, traded species are often associated with widespread Asian bird cultural traditions, such as prayer release, song competitions, or bird‐keeping (Severinghaus & Chi, 1999). Therefore, these species for sale should be more likely to originate from neighboring areas.

Finally, if geographical factors were important, we would expect alien species to be more likely sourced from neighboring countries of the studied market because these species should be more available for trade (Chng et al., 2015; Edmunds et al., 2011; Gilbert et al., 2012; Nijman, 2010; Shepherd, 2006). Thus, we should not expect similarities in species shared among the three markets due to their disparate locations.

## 2 METHODS

### 2.1 Data collection

Data for the traded alien bird species in the Iberian market (Spain and Portugal), including the continent and the associated Balearic, Canary, Madeira, and Azores archipelagos, were obtained from Table S3 in Abellán et al. (2016). The authors surveyed a variety of sources to record alien species kept in captivity in public and private facilities up to 2012. Species alien to the region were classified according to their geographical range rather than political borders, so species native to continental Spain or Portugal were classified as alien when they were transported to its islands or vice versa.

We compiled information on alien species recorded in captivity in Taiwan from 1991 to 2012, from pet shop surveys conducted after the application of the Wildlife Conservation Act in 1989 (Chi, 1995; Chi et al., 1991; Shieh et al., 2006; Su et al., 2014; Wong et al., 2012). To this list, we added data from a checklist of alien species imported into Taiwan from Yen (2011), which includes imported species from 2009 to 2011. The checklist information was collated from the stock records and invoices from bird traders, Internet trade, web databases, published lists, previous nationally funded projects, and zoo species checklists.

Alien species in captivity in Australia were recorded for the period 2003–2013 (Vall‐llosera & Cassey, 2017) and mainly collected from two aviculturist magazines, Australian Birdkeeper (2011) and Australian Aviculture, and a survey published by the National Finch and Softbill Association (The Avicultural Society of Australia Inc., 2011; The Queensland Finch Society Inc., 2012; The United Bird Societies of South Australia Inc., 2003). We did not exclude species that were illegally traded.

The taxonomy of species followed Jetz et al. (2012). The geographic origin of alien species present in markets was assessed using the biogeographical realms defined in Olson et al. (2001), assigning species to the realm in which the largest part of their native range fell (Dyer et al., 2016; Orme et al., 2006).

### 2.2 Analyses

We first determined whether the taxonomy (number of species per Order and Family) and origin (i.e., the realm of origin) of traded alien species at each market was significantly different than expected under a random expectation of the world's extant species. Specifically, we used a randomization approach based on Ref. (Su et al., 2014). Simulations involved picking the number of alien bird species observed in each market from all the world's extant species (*n* = 9993) listed in Jetz et al. (2012) without replacement. For comparisons of alien species between two markets, any species native to either of those two countries were removed from that analysis. Simulations were run for 10,000 iterations to obtain all the numbers of randomly chosen species per Family, Order, and origin. Then, we estimated whether our observations were significantly different from those expected at random. We estimated significance by a two‐tailed t test with the significance level (*α*) adjusted by sequential Bonferroni correction for multiple comparisons, at 0.0015 and 0.00024 for comparisons at the Order and Family level (40 orders and 194 families), respectively, and tested for the biogeographic realms of species origin (eight realms, significance level *α* = 0.0062, adjusted by sequential Bonferroni correction). We repeated our simulations constraining the Order and Family membership to test for overlap in the identities of traded species among the different markets (i.e., the Iberian and Taiwanese markets, the Iberian and Australian markets, the Australian and Taiwanese markets, and the overlap across all markets combined). Finally, we tested where these commonly traded species were likely to be derived from, given the observed numbers of species shared by each market. All analyses were performed in the R software environment for statistical and graphical computing version 4.0.4 (R Core Team, 2021).

## 3 RESULTS

A total of 1166 alien bird species, accounting for more than 10% of the world's bird species, were identified as traded in our study areas. There were 1022 alien bird species from 29 Orders and 75 Families recorded in the Iberian market, while the numbers were lower in Taiwan (409 species, 20 Orders, 53 Families) and Australia (185 species, 11 Orders, 24 Families). Only six of the 40 extant avian Orders and 10 of the 194 extant avian Families were overrepresented in the three markets. Most of the traded species belonged to the Orders Passeriformes (passerines) and Psittaciformes (parrots) (Table 1), specifically to the Families Estrildidae and Psittacidae, respectively, but also to the Families Columbidae and Anatidae (Table 1b). Except for the Order Passeriformes, for which species were traded in significantly lower numbers than expected based on its richness (*α* = 0.0015), all the other significantly traded Orders and Families had a higher number of traded species than expected at random (*α* = 0.00024) (Table 1).

**TABLE 1:** Number of alien species per (a) Order and (b) Family overrepresented in the Iberian, Australian, and Taiwanese markets, and shared patterns among them

(a)														
Order	Total species						Overlapping species							
	Iberia		Australia		Taiwan		Iberia and Australia		Iberia and Taiwan		Australia and Taiwan		Iberia, Taiwan, and Australia	
	(*n* = 922)		(*n* = 160)		(*n* = 330)		(*n* = 158)		(*n* = 259)		(*n* = 104)		(*n* = 102)	
	Observed	Expected	Observed	Expected	Observed	Expected	Observed	Expected	Observed	Expected	Observed	Expected	Observed	Expected
Passeriformes	275	610	77	127	177	235	59	11	74	25	28	4	27	0
		(554–659)		(101–151)		(193–270)		(2–25)		(11–46)		(0–15)		(0–5)
Psittaciformes	241	36	63	7	145	14	63	1	143	1	53	0	53	0
		(17–57)		(1–19)		(3–29)		(0–6)		(0–8)		(0–5)		(0–2)
Galliformes	102	29	18	6			17	0	16	1	12	0	11	0
		(12–48)		(0–16)				(0–4)		(0–8)		(0–3)		(0–2)
Columbiformes	91	31					9	0	9	1	4	0	4	0
		(14–51)						(0–5)		(0–7)		(0–3)		(0–2)
Anseriformes	89	16					9	0	11	0	5	0	5	0
		(4–31)						(0–4)		(0–5)		(0–2)		(0–2)
Accipitriformes	47	26					1	0						
		(8–43)						(0–0)						
Strigiformes	34	21												
		(13–30)												
Bucerotiformes	18	7												
		(2–16)												
Musophagiformes	14	2												
		(0–8)												
Sphenisciformes	8	2			4	1			3	0			2	0
		(0–7)				(0–3)				(0–2)				(0–1)
Phoenicopteriformes	3	0	2	0	4	0			3	0	2	0		
		(0–2)		(0–1)		(0–3)				(0–2)		(0–1)		

(b)														
Family	Total species						Overlapping species							
	Iberia		Australia		Taiwan		Iberia and Australia		Iberia and Taiwan		Australia and Taiwan		Iberia, Taiwan, and Australia	
	(n = 756)		(n = 130)		(n = 196)		(n = 141)		(n = 224)		(n = 84)		(n = 96)	
	Observed	Expected	Observed	Expected	Observed	Expected	Observed	Expected	Observed	Expected	Observed	Expected	Observed	Expected
Psittacidae	241	36 (17–59)	63	7 (0–18)	145	14 (3–28)	63	0 (0–6)	143	1 (0–10)	53	0 (0–3)	53	0 (0–2)
Columbidae	91	31 (15–52)					9	0 (0–5)					4	0 (0–2)
Anatidae	87	16 (5–32)					9	0 (0–3)	11	0 (0–5)	5	0 (0–3)	5	0 (0–2)
Estrildidae	78	14 (3–30)	35	3 (0–12)	27	5 (0–15)	30	0 (0–3)	23	0 (0–5)	12	0 (0–2)	12	0 (0–2)
Phasianidae	71	18 (6–35)	14	3 (0–13)			13	0 (0–4)	12	1 (0–5)	10	0 (0–3)	9	0 (0–1)
Accipitridae	43	25 (4–41)												
Fringillidae	41	17 (5–33)	18	3 (0–11)			13	0 (0–4)	9	1 (0–5)	4	0 (0–3)	4	0 (0–1)
Sturnidae	30	11 (2–26)			19	4 (0–13)			11	0 (0–5)				
Ploceidae	25	11 (2–23)					4	0 (0–3)	6	0 (0–4)			2	0 (0–1)
Cracidae	19	5 (0–16)												
Bucerotidae	16	5 (0–15)												
Musophagidae	14	2 (0–10)												
Chloropseidae					5	0 (0–4)								
Gruidae									6	0 (0–2)				
Phoenicopteridae									3	0 (0–2)				
Spheniscidae													2	0 (0–1)
Emberizidae													3	0 (0–2)
Odontophoridae													2	0 (0–1)

Note

*n*: the total numbers of species among the Orders or Families that were significantly differ from random expectation. Observed: the numbers of traded alien species from each Order or Family. Expected: median expectation over the 10,000 simulations of the randomization test. In brackets: 99.92th and 0.08th percentiles, indicating the values of upper and lower limits of significance (*α* = 0.0015 and *α* = 0.00024, for Order and Family, respectively, adjusted by sequential Bonferroni correction).

Alien species traded in Australian market have 88% overlap with those of the Iberian market, strongly selecting species within the Families Psittacidae, Estrildidae, Phasianidae, and Fringillidae. Alien birds present in the Iberian and Australian markets originated mainly from the Neotropics (27% and 32%, respectively) and Afrotropics (26% and 27%, respectively), while those traded in Taiwan were mainly from the Indo‐Malayan (23%) realm (Table 2). Iberia and Australia traded more alien species from the Afrotropics than expected, while Taiwan followed the same pattern but with species also from the Indo‐Malayan and Palearctic realms (Table 2a).

**TABLE 2:** Geographic origin of (a) alien species traded at each of the markets, and (b) for those overlapped among them

(a)						
Biogeographic Realms	Iberia		Australia		Taiwan	
	(*n* = 1022)		(*n* = 185)		(*n* = 409)	
	Observed	Expected	Observed	Expected	Observed	Expected
Afrotropics	269*	195 (163–228)	51*	35 (21–50)	77	78 (58–100)
Antarctic	5	4 (0–10)	2	1 (0–4)	3	2 (0–6)
Australasia	172	157 (128–188)	0	28 (16–42)	75	63 (44 −83)
Indo‐Malayan	131	135 (108–164)	37	24 (13–38)	97*	54 (37–73)
Nearctic	51	49 (33–67)	5	9 (2 −18)	8**	20 (9–32)
Neotropics	281**	361 (323–399)	60	65 (47–83)	89**	145 (119–170)
Oceania	5**	19 (9 −32)	2	3 (0–9)	1**	8 (2–16)
Palearctic	108	100 (76–125)	28	18 (8–30)	59*	40 (24–57)

(b)								
Biogeographic Realms	Iberia and Australia		Australia and Taiwan		Iberia and Taiwan		All the markets	
	(*n* = 163)		(*n* = 107)		(*n* = 284)		(*n* = 104)	
	Observed	Expected	Observed	Expected	Observed	Expected	Observed	Expected
Afrotropics	45	31 (18–45)	27	20 (10–32)	63	54 (36–73)	26	20 (10–31)
Antarctic	2	1 (0–4)	2	0 (0–3)	2	1 (0–5)	2	0 (0–3)
Australasia	0	25 (13–38)	0	16 (7–27)	69*	43 (28–61)	0	16 (7–27)
Indo‐Malayan	32	21 (11–34)	19	14 (5–25)	44	38 (23–54)	19	14 (6–24)
Nearctic	5	8 (2 −16)	4	5(0–12)	5	14 (5–25)	4	5 (0–12)
Neotropics	60	58 (42–75)	44	38 (25–52)	82	100 (79–122)	44	37 (24 −50)
Oceania	0	3 (0–9)	0	2 (0–7)	1	5 (0–13)	0	2 (0–7)
Palearctic	19	16 (7–27)	11	10 (3–19)	18	28 (15–42)	9	10 (3–19)

(c)						
Biogeographic Realms (parrots)	Iberia		Australia		Taiwan	
	(*n* = 241)		(*n* = 63)		(*n* = 145)	
	Observed	Expected	Observed	Expected	Observed	Expected
Afrotropics	20	16 (9–21)	10*	4 (0–9)	16*	9 (3–15)
Antarctic	0	NA	0	NA	0	NA
Australasia	91	94 (83–106)	0	25 (15–34)	55	57 (45–69)
Indo‐Malayan	16	20 (13–26)	7	5 (1–11)	8	12 (6–19)
Nearctic	2	1 (0–2)	0	0 (0–2)	0	1 (0–2)
Neotropics	109	102 (91–113)	46*	27 (17 −37)	65	61 (50–74)
Oceania	3	7 (3 −10)	0	2 (0–6)	1	4 (0–8)
Palearctic	0	NA	0	NA	0	NA

(d)								
Biogeographic Realms (parrots)	Iberia and Australia		Australia and Taiwan		Iberia and Taiwan		All the markets	
	(*n* = 63)		(*n* = 53)		(*n* = 143)		(*n* = 53)	
	Observed	Expected	Observed	Expected	Observed	Expected	Observed	Expected
Afrotropics	10*	4 (0–9)	9*	3 (0–8)	16*	9 (3–15)	9*	3 (0–8)
Antarctic	0	NA	0	NA	0	NA	0	NA
Australasia	0	25 (15–35)	0	21 (12–30)	54	56 (44–69)	0	21 (12–30)
Indo‐Malayan	7	5 (1–11)	5	4 (0–10)	7	12 (6 −19)	5	4 (0–10)
Nearctic	0	0 (0–2)	0	0 (0–2)	0	1 (0–2)	0	0 (0–2)
Neotropics	46*	27 (17–37)	39*	22 (14–32)	65	60 (48–73)	39*	22 (13–32)
Oceania	0	2 (0 −6)	0	1 (0–5)	1	4 (0–8)	0	1 (0 −5)
Palearctic	0	NA	0	NA	0	NA	0	NA

(e)						
Biogeographic Realms (passerines)	Iberia		Australia		Taiwan	
	(*n* = 275)		(*n* = 77)		(*n* = 177)	
	Observed	Expected	Observed	Expected	Observed	Expected
Afrotropics	108*	56 (39–73)	34*	16 (7–26)	41	36 (22–51)
Antarctic	0	0 (1–0)	0	0 (0–1)	0	0 (0–1)
Australasia	30	41 (26 −58)	0	11 (4–21)	12**	26 (14–40)
Indo‐Malayan	41	36 (22–51)	18	10 (3–19)	67*	23 (12–36)
Nearctic	10	12 (4–23)	1	3 (0–9)	2	8 (2–16)
Neotropics	48**	99 (78–120)	9**	28 (17–39)	13**	64 (47–81)
Oceania	0	4 (0–11)	1	1 (0–5)	0	3 (0–8)
Palearctic	38	26 (14–40)	14	7 (1–15)	42*	16 (7–28)

(f)								
Biogeographic Realms (passerines)	Iberia and Australia		Australia and Taiwan		Iberia and Taiwan		All the markets	
	(*n* = 59)		(*n* = 28)		(*n* = 74)		(*n* = 27)	
	Observed	Expected	Observed	Expected	Observed	Expected	Observed	Expected
Afrotropics	29*	12 (6–18)	13*	6 (2–10)	30*	15 (9–22)	13*	5 (2–10)
Antarctic	0	0	0	0	0	0	0	0
Australasia	0	9 (4–14)	0	4 (1–8)	8	11(6–17)	0	4 (1–8)
Indo‐Malayan	14*	8 (3–13)	8*	4 (1–7)	22*	10 (4 −16)	8*	3 (0–7)
Nearctic	1	3 (0–6)	0	1 (0–4)	0	3 (0–7)	0	1 (0–4)
Neotropics	9**	21 (14–29)	4**	10 (5–15)	7**	27 (19–35)	4**	10 (5–15)
Oceania	0	1 (0–3)	0	0 (0–2)	0	1 (0–4)	0	0 (0 −2)
Palearctic	6	5 (2–10)	3	2 (0–6)	7	7 (3–12)	2	2 (0–6)

Note

Alien parrot species are separately shown in (c) and (d). For parrot species in each market, we only considered the six biogeographic realms with native parrot species. Alien passerine species are shown in (e) and (f). Observed: the numbers of traded species derived from each realm. Expected: median expectation over the 10,000 simulations of the randomization test. In brackets: 99.69th and 0.31th percentiles, indicating the values of upper and lower limits of significance (*α* = 0.0062, adjusted by sequential Bonferroni correction). Asterisks (*) are denoted if the observed number is significantly greater (*) or smaller (**) than expected from the randomization test.

**FIGURE 1:**
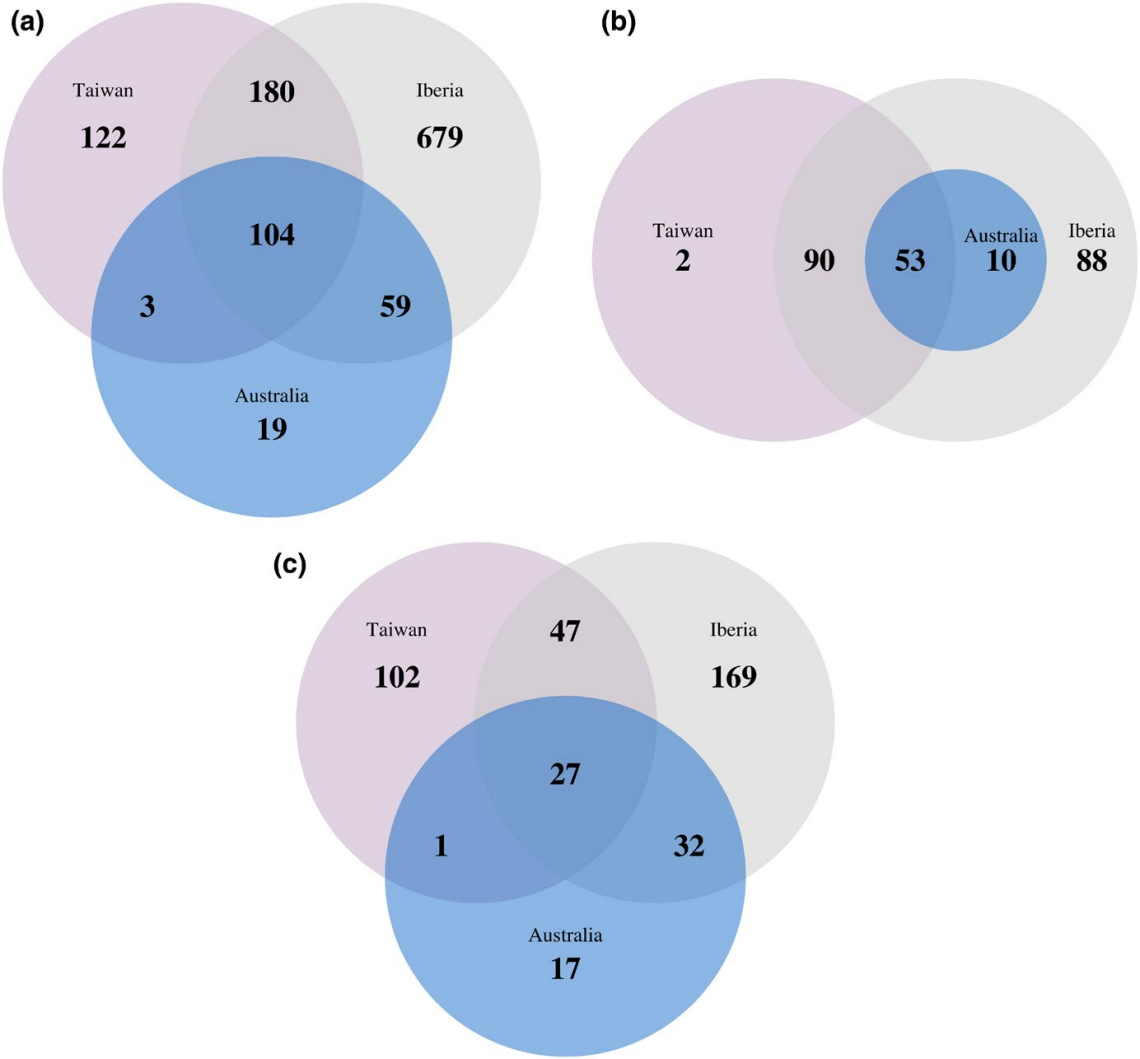
The numbers of (a) all alien, (b) alien parrot, and (c) alien passerine species for sale across the Iberian, Australian, and Taiwanese bird trade markets

**FIGURE 2:**
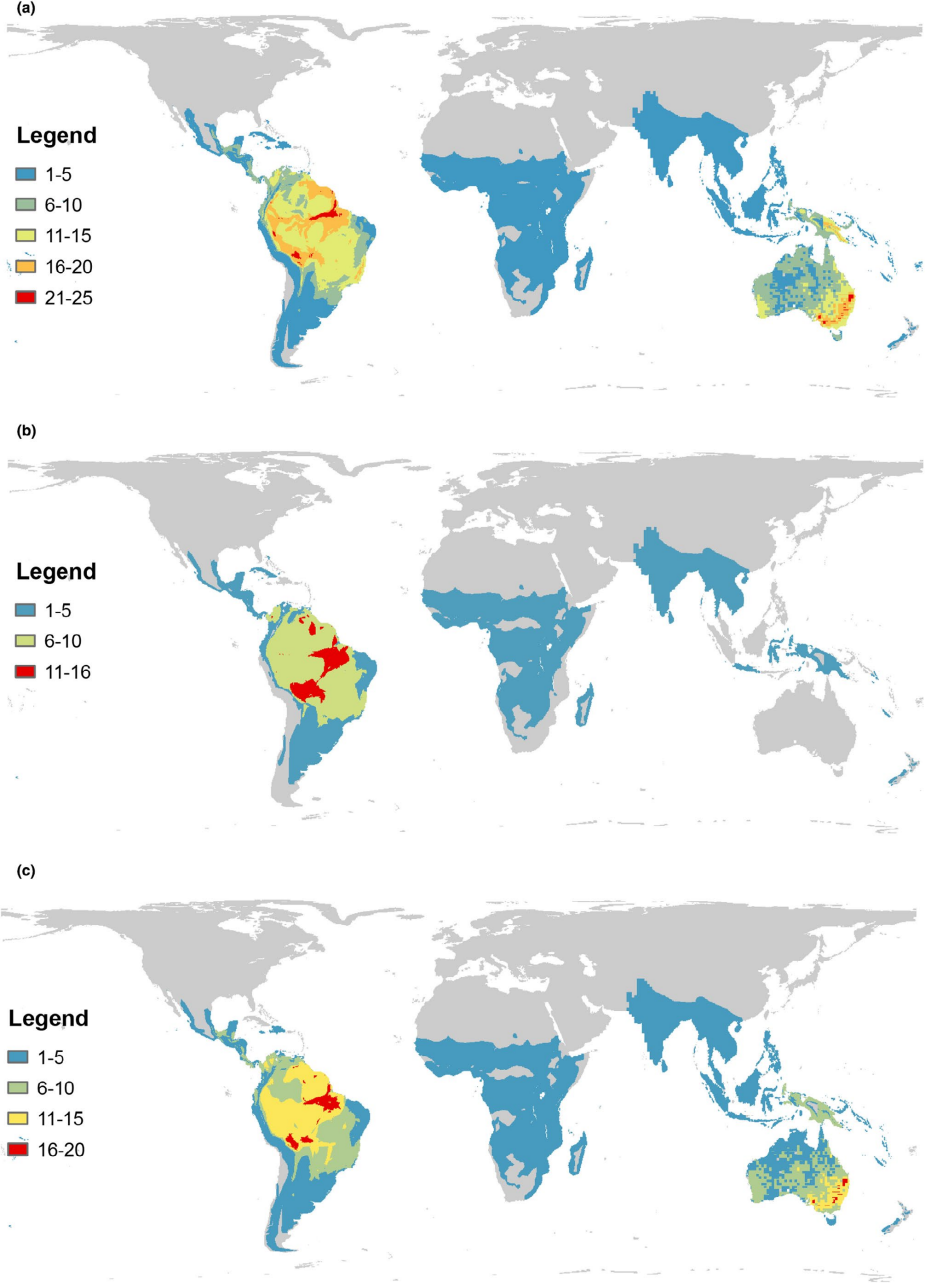
Native geographic ranges for alien parrots traded to (a) the Iberian (*n* = 236), (b) Australian (*n* = 57), and (c) Taiwanese (*n* = 140) bird markets. Maps were constructed by adding all native range of the alien parrot species traded in each region

In terms of the numbers of species overlapping, the Iberian and Taiwanese markets shared the largest number of alien bird species (284 species, 35 Families, 18 Orders), compared with the Iberian and Australian (163 species, 21 Families, 10 Orders), and the Australian and Taiwanese markets (107 species, 17 Families, 8 Orders) (Figure 1). Although the number of species from the Neotropics was very important in all markets, the observed numbers were lower than expected in the Iberian and Taiwanese markets (Table 2). Comparisons between markets revealed that there were more shared species from Australasia than expected between the Iberian and Taiwanese markets (Table 2b). In particular, alien parrots commonly traded in the Iberian and Taiwanese markets mostly originated from the Neotropical and Australasian realms, while most alien parrots traded in Australia were Neotropical species (Figure 2).

A total of 104 species from 16 Families and 8 Orders were traded as aliens in all three markets (Figure 1). This selection of species within particular Orders and Families results in a marked overlap between the three study markets. Psittaciformes and Passeriformes (specifically, Families Psittacidae and Estrildidae) were the taxa with the greatest numbers of species common to the three markets (Table 1). There were also some Families exclusively traded in some particular markets, such as the Accipitridae (raptors), Cracidae (guans), Bucerotidae (hornbills), and Musophagidae (turacos), which were traded more than expected only in the Iberian market, or Chloropseidae (leafbirds), which were overrepresented only in the Taiwanese market. There were no overrepresented Families exclusive to the Australian market. 245 parrot species were identified for sale in our study, almost 70% of all extant parrot species. Parrot species from the Afrotropical and Neotropical regions were, in general, more shared among the three markets than expected by chance (Table 2d).

## 4 DISCUSSION

Wildlife trade is one of the main sources of current biological invasions worldwide (Lockwood et al., 2019). Thus, understanding which drivers are acting at the earliest stages of the invasion process (i.e., in the pool of species available for sale) can be useful to identify and prevent further invasive alien species. Birds are one of the most commonly traded taxonomic groups, with ca. 4000 of both wild‐caught and captive‐bred species mainly sold and kept as pets (BirdLife International, 2015). However, not all species are equally traded. International and regional trade regulations have limited species choices in legal bird markets (Cardador et al., 2019), and aspects such as species attractiveness (Romero‐Vidal et al., 2020), availability (e.g., species abundance in the wild or from captive breeding), or life‐history can be important in explaining which species are selected over others (Su, Cassey, Vall‐llosera, et al., 2015; Vall‐Llosera & Su, 2018). Here, using a straightforward simulation approach, we explored the role of cultural, historical, and geographic factors in affecting the pool of alien bird species presented in three distant markets, located on three separate continents.

Our first prediction was that cultural and historical factors could be driving the species composition of bird markets, so that the Australian and Iberian ones should be similar to each other in their taxonomic composition and realm of origin of the species traded. Our study found that alien species traded in the Australian market highly overlapped with those of the Iberian market. The Australian and Iberian markets had more alien bird species in common compared with the Australian and Taiwanese markets. However, contrary to our prediction, the Iberian and Taiwanese markets shared the largest number of alien bird species compared with the Iberian and Australian markets. The Iberian and Australian markets showed a strong overrepresentation of certain avian Families, such as Phasianidae, Anatidae, and Columbidae, which have a long history of trade in the European culture for their use as food and hunting, being deliberately introduced by European settlers in Australia and New Zealand (Duncan et al., 2006). However, such demand has been reduced during recent decades and today trades include ornamental birds (Abellán et al., 2016), especially passerines and parrots for their use as cage birds and pets. Thus, species derived from the Psittaciformes and Passeriformes were found dominantly traded in the two markets. Alien birds simultaneously presented in the Iberian and Australian markets were mostly from the Neotropics and Afrotropics, reflecting the importance of transportation routes from past colonies (Cassey et al., 2015). Conversely, the Taiwanese market was mainly supplied with species from the Indo‐Malayan realm, as predicted, supporting a role for cultural and historical drivers of alien species presence in bird markets.

Our expectation regarding the role of geographic factors in determining the composition of alien species in the markets was partially supported. Although there was a high overlap in alien species presented in the three distant markets, there were also certain taxa that were exclusive to certain markets. For instance, the Taiwanese market was mostly supplied with alien species from nearby regions, which may be related to the common interests toward certain bird groups shared with neighboring countries, such as leafbirds (Chloropseidae), which were significantly overrepresented in this market only. This is notable as leafbirds, which are kept for their attractive plumage and songs, are expensive due to their being traded illegally, and also because they have a low survival rate while being traded (Alacs & Georges, 2008; Wyatt, 2009).

The number of alien species in the Iberian market was twice the number traded in Taiwan and almost five times higher than in Australia. The high richness of species traded in the Iberian market could be a consequence of a more complete assessment of the pet trade in this area or the inclusion of islands in our dataset. However, it is more likely that the long tradition of captive breeding, combined with the role of Iberia as the main importer of alien birds for Europe, and the lack of wild‐caught bird trade restrictions until 2005, would have been responsible for the importation of large numbers of alien species in this area (Abellán et al., 2016; Cardador et al., 2019). Although there is also a long history of alien bird trade in Taiwan, this country may have received a lower number of species from distant areas because of transportation restrictions with China in the 20^th^ century and its insularity. Importantly, the bird market in Taiwan is normally supplied with native species poached locally (Severinghaus & Chi, 1999; Su et al., 2014), something that is much rarer in Iberian countries and Australia.

Despite the fact that we found strong support on the influences of cultural and historical factors in species composition, our results support the rather weak influence of geographic factors in determining the pool of alien species in European, Australian, and Asian markets. Globalization and the possibility of moving species across the world have played a major role in homogenizing markets at large spatial scales, such as those explored here. Moreover, the inclusion of several species into CITES I and II Appendices may have also restricted the availability of species to be traded, which have now restricted quotas or are scarce and illegally traded. Finally, bans applied in the United States (1992) and European Union (2005) on the international trade of wild‐caught species have caused the redirection of birds to different markets, such as those in Asia (Cardador et al., 2017). Restrictions in species importation in Australia may have resulted in a relatively lower number of alien traded species (Cardador et al., 2017). Thus, factors operating at a global scale may now be more important in determining the initial pool of alien species available for introduction in distant areas than in the past. Although at first glance it can seem easier to manage local markets, our results support the idea that large‐scale restrictions can be beneficial to preserve local biodiversity, simply by reducing the probability of introducing invasive species (Cardador et al., 2017; Reino et al., 2017).

Despite differences among markets, our study identified a strong commonality in the composition of traded species across Western and Eastern markets. Some species were heavily traded and shared among the markets, which suggests that they were likely to be frequently transported. This, in turn, may result in the introduction into new areas of a relatively limited set of species (Cassey et al., 2018), which may positively affect their invasion success (Abellán et al., 2017; Cassey et al., 2004; Duncan et al., 2001; Signorile et al., 2014; Su et al., 2015). We found that all three markets included the two most invasive parrot species in the world, the rose‐ringed parakeet (*Psittacula krameria*) and the monk parakeet (*Myiopsitta monachus*), despite their numerous recorded impacts (Turbé et al., 2017; Vall‐llosera et al., 2017). Finally, our results provide insights into the role played by anthropological drivers in configuring the pool of potentially invasive species at the earliest stage of the invasion process, when they are transported to a new region, and how globalization may be influencing them, facilitating the movement of species and the homogenization of species traded worldwide.

## CONFLICT OF INTEREST

None of the authors have any competing interests.

## AUTHOR CONTRIBUTIONS

**Shan Su:** Conceptualization (lead); Data curation (equal); Formal analysis (lead); Funding acquisition (equal); Investigation (lead); Methodology (lead); Project administration (equal); Resources (equal); Software (equal); Supervision (lead); Validation (equal); Visualization (lead); Writing–original draft (lead); Writing–review & editing (equal). **Miquel Vall‐llosera:** Conceptualization (equal); Data curation (equal); Formal analysis (equal); Funding acquisition (equal); Investigation (equal); Methodology (equal); Project administration (equal); Resources (equal); Software (equal); Supervision (lead); Validation (equal); Visualization (equal); Writing–original draft (equal); Writing–review & editing (equal). **Phillip Cassey:** Conceptualization (equal); Data curation (equal); Formal analysis (equal); Funding acquisition (equal); Investigation (equal); Methodology (equal); Project administration (equal); Resources (equal); Software (equal); Supervision (lead); Validation (equal); Visualization (equal); Writing–original draft (equal); Writing–review & editing (equal). **Tim M. Blackburn:** Conceptualization (equal); Data curation (equal); Formal analysis (equal); Funding acquisition (equal); Investigation (equal); Methodology (equal); Project administration (equal); Resources (equal); Software (equal); Supervision (lead); Validation (equal); Visualization (equal); Writing–original draft (equal); Writing–review & editing (equal). **Martina Carrete:** Conceptualization (equal); Data curation (supporting); Formal analysis (equal); Funding acquisition (equal); Investigation (equal); Methodology (equal); Project administration (equal); Resources (equal); Software (equal); Supervision (equal); Validation (equal); Visualization (equal); Writing–original draft (equal); Writing–review & editing (equal). **José L. Tella:** Conceptualization (equal); Data curation (equal); Formal analysis (equal); Funding acquisition (equal); Investigation (equal); Methodology (equal); Project administration (equal); Resources (equal); Software (equal); Supervision (lead); Validation (equal); Visualization (equal); Writing–original draft (equal); Writing–review & editing (equal).

## Data Availability

The data that support the findings of this study are openly available in Dryad at https://doi.org/10.5061/dryad.7wm37pvtv.

## References

[ece38397-bib-0001] Abellán, P. , Carrete, M. , Anadón, J. D. , Cardador, L. , & Tella, J. L. (2016). Non‐random patterns and temporal trends (1912–2012) in the transport, introduction and establishment of exotic birds in Spain and Portugal. Diversity and Distributions, 22(3), 263–273. 10.1111/ddi.12403

[ece38397-bib-0002] Abellán, P. , Tella, J. L. , Carrete, M. , Cardador, L. , & Anadón, J. D. (2017). Climate matching drives spread rate but not establishment success in recent unintentional bird introductions. Proceedings of the National Academy of Sciences of the United States of America, 114(35), 9385–9390. 10.1073/pnas.1704815114 28784783 PMC5584426

[ece38397-bib-0003] Alacs, E. , & Georges, A. (2008). Wildlife across our borders: A review of the illegal trade in Australia. Australian Journal of Forensic Sciences, 40(2), 147–160. 10.1080/00450610802491382

[ece38397-bib-0004] Birdkeeper, A. (2011). Classifieds. Australian Birdkeeper.

[ece38397-bib-0005] BirdLife International (2015). Statement during the Ministerial Segment at the International Conference to Develop a Common Strategy to Combat Illegal Wildlife Trade. BirdLife International.

[ece38397-bib-0006] Blackburn, T. M. , & Cassey, P. (2007). Patterns of non‐randomness in the exotic avifauna of Florida. Diversity and Distributions, 13(5), 519–526. 10.1111/j.1472-4642.2007.00358.x

[ece38397-bib-0007] Blackburn, T. M. , & Duncan, R. P. (2001). Establishment patterns of exotic birds are constrained by non‐random patterns in introduction. Journal of Biogeography, 28(7), 927–939. 10.1046/j.1365-2699.2001.00597.x

[ece38397-bib-0008] Blackburn, T. M. , Lockwood, J. L. , & Cassey, P. (2009). Avian invasions: The ecology and evolution of exotic birds. Oxford University Press. http://public.eblib.com/EBLPublic/PublicView.do?ptiID=679349

[ece38397-bib-0009] Blackburn, T. M. , Pyšek, P. , Bacher, S. , Carlton, J. T. , Duncan, R. P. , Jarošík, V. , Wilson, J. R. U. , & Richardson, D. M. (2011). A proposed unified framework for biological invasions. Trends in Ecology & Evolution, 26(7), 333–339. 10.1016/j.tree.2011.03.023 21601306

[ece38397-bib-0010] Bush, E. R. , Baker, S. E. , & Macdonald, D. W. (2014). Global trade in exotic pets 2006–2012: Exotic pet trade. Conservation Biology, 28(3), 663–676. 10.1111/cobi.12240 24661260

[ece38397-bib-0011] Cardador, L. , Carrete, M. , Gallardo, B. , & Tella, J. L. (2016). Combining trade data and niche modelling improves predictions of the origin and distribution of non‐native European populations of a globally invasive species. Journal of Biogeography, 43(5), 967–978. 10.1111/jbi.12694

[ece38397-bib-0012] Cardador, L. , Lattuada, M. , Strubbe, D. , Tella, J. L. , Reino, L. , Figueira, R. , & Carrete, M. (2017). Regional bans on wild‐bird trade modify invasion risks at a global scale: Trade bans and invasion risk. Conservation Letters, 10(6), 717–725. 10.1111/conl.12361

[ece38397-bib-0013] Cardador, L. , Tella, J. L. , Anadón, J. D. , Abellán, P. , & Carrete, M. (2019). The European trade ban on wild birds reduced invasion risks. Conservation Letters, 12(3), e12631. 10.1111/conl.12631

[ece38397-bib-0014] Carrete, M. , & Tella, J. (2008). Wild‐bird trade and exotic invasions: A new link of conservation concern? Frontiers in Ecology and the Environment, 6(4), 207–211. 10.1890/070075

[ece38397-bib-0015] Carrete, M. , & Tella, J. L. (2015). Rapid loss of antipredatory behaviour in captive‐bred birds is linked to current avian invasions. Scientific Reports, 5, 18274. 10.1038/srep18274 26667185 PMC4678868

[ece38397-bib-0016] Cassey, P. , Blackburn, T. M. , Sol, D. , Duncan, R. P. , & Lockwood, J. L. (2004). Global patterns of introduction effort and establishment success in birds. Proceedings of the Royal Society B: Biological Sciences, 271(Suppl_6), S405–S408. 10.1098/rsbl.2004.0199 PMC1810115 15801588

[ece38397-bib-0017] Cassey, P. , Delean, S. , Lockwood, J. L. , Sadowski, J. S. , & Blackburn, T. M. (2018). Dissecting the null model for biological invasions: A meta‐analysis of the propagule pressure effect. PLOS Biology, 16(4), e2005987. 10.1371/journal.pbio.2005987 29684017 PMC5933808

[ece38397-bib-0018] Cassey, P. , Vall‐llosera, M. , Dyer, E. , & Blackburn, T. M. (2015). The biogeography of avian invasions: History, accident and market trade. In J. Canning‐Clode & F. Paiva (Eds.), Biological invasions in changing ecosystems: Vectors, ecological impacts, management and predictions (pp. 38–54). De Gruyter Open.

[ece38397-bib-0019] Chi, W. L. (1995). An investigation report on pet bird trade in Taiwan (pp. 46). Green Consumer's Foundation.

[ece38397-bib-0020] Chi, W. L. , Chen, A.‐L. , Yiau, H.‐C. , Wang, S.‐Y. , & Zhong, Z.‐S. (1991). The sale status of wild birds in Taipei City. Wildbirds, 2, 29–36.

[ece38397-bib-0021] Chng, S. C. L. , Eaton, J. A. , Krishnasamy, K. , Shepherd, C. R. , & Nijman, V. (2015). TRAFFIC report: In the market for extinction: An inventory of Jakarta's bird. TRAFFIC.

[ece38397-bib-0022] Duncan, R. P. , Blackburn, T. M. , & Cassey, P. (2006). Factors affecting the release, establishment and spread of introduced birds in New Zealand. In R. B. Allen & W. G. Lee (Eds.), Biological invasions in New Zealand (Vol. 186, pp. 137–154). Springer.

[ece38397-bib-0023] Duncan, R. P. , Bomford, M. , Forsyth, D. M. , & Conibear, L. (2001). High predictability in introduction outcomes and the geographical range size of introduced Australian birds: A role for climate. Journal of Animal Ecology, 70(4), 621–632. 10.1046/j.1365-2656.2001.00517.x

[ece38397-bib-0024] Dyer, E. E. , Cassey, P. , Redding, D. W. , Collen, B. , Franks, V. , Gaston, K. J. , Jones, K. E. , Kark, S. , Orme, C. D. L. , & Blackburn, T. M. (2017). The global distribution and drivers of alien bird species richness. PLOS Biology, 15(1), e2000942. 10.1371/journal.pbio.2000942 28081142 PMC5230740

[ece38397-bib-0025] Dyer, E. E. , Franks, V. , Cassey, P. , Collen, B. , Cope, R. C. , Jones, K. E. , Şekercioğlu, Ç. H. , & Blackburn, T. M. (2016). A global analysis of the determinants of alien geographical range size in birds: Geographical range size in alien birds. Global Ecology and Biogeography, 25, 1346–1355. 10.1111/geb.12496

[ece38397-bib-0026] Eaton, J. A. , Nguyen, M. D. T. , Willemsen, M. , Lee, J. , & Chng, S. C. L. (2017). Caged in the city: An inventory of birds for sale in Ha Noi and Ho Chi Minh City, Viet Nam. TRAFFIC, Southeast Asia Regional Office.

[ece38397-bib-0027] Edelaar, P. , Roques, S. , Hobson, E. A. , Gonçalves da Silva, A. , Avery, M. L. , Russello, M. A. , Senar, J. C. , Wright, T. F. , Carrete, M. , & Tella, J. L. (2015). Shared genetic diversity across the global invasive range of the monk parakeet suggests a common restricted geographic origin and the possibility of convergent selection. Molecular Ecology, 24(9), 2164–2176. 10.1111/mec.13157 25873354

[ece38397-bib-0028] Edmunds, K. , Roberton, S. I. , Few, R. , Mahood, S. , Bui, P. L. , Hunter, P. R. , & Bell, D. J. (2011). Investigating Vietnam's ornamental bird trade: Implications for transmission of zoonoses. EcoHealth, 8(1), 63–75. 10.1007/s10393-011-0691-0 21809163

[ece38397-bib-0029] Gilbert, M. , Sokha, C. , Joyner, P. H. , Thomson, R. L. , & Poole, C. (2012). Characterizing the trade of wild birds for merit release in Phnom Penh, Cambodia and associated risks to health and ecology. Biological Conservation, 153, 10–16. 10.1016/j.biocon.2012.04.024

[ece38397-bib-0030] Heinrich, S. , Ross, J. V. , Gray, T. N. E. , Delean, S. , Marx, N. , & Cassey, P. (2020). Plight of the commons: 17 years of wildlife trafficking in Cambodia. Biological Conservation, 241, 108379. 10.1016/j.biocon.2019.108379

[ece38397-bib-0031] Jepson, P. , & Ladle, R. J. (2005). Bird‐keeping in Indonesia: Conservation impacts and the potential for substitution‐based conservation responses. Oryx, 39(4), 442–448. 10.1017/S0030605305001110

[ece38397-bib-0032] Jeschke, J. M. , & Strayer, D. L. (2006). Determinants of vertebrate invasion success in Europe and North America. Global Change Biology, 12(9), 1608–1619. 10.1111/j.1365-2486.2006.01213.x

[ece38397-bib-0033] Jetz, W. , Thomas, G. H. , Joy, J. B. , Hartmann, K. , & Mooers, A. O. (2012). The global diversity of birds in space and time. Nature, 491(7424), 444–448. 10.1038/nature11631 23123857

[ece38397-bib-0034] Lee, T. W. , & Shieh, B. S. (2005). Pet sales of exotic estrildid birds in relation to the field‐records in Taiwan. Endemic Species Research, 7, 1–12.

[ece38397-bib-0035] Lockwood, J. L. , Welbourne, D. J. , Romagosa, C. M. , Cassey, P. , Mandrak, N. E. , Strecker, A. , Leung, B. , Stringham, O. C. , Udell, B. , Episcopio‐Sturgeon, D. J. , Tlusty, M. F. , Sinclair, J. , Springborn, M. R. , Pienaar, E. F. , Rhyne, A. L. , & Keller, R. (2019). When pets become pests: The role of the exotic pet trade in producing invasive vertebrate animals. Frontiers in Ecology and the Environment, 17(6), 323–330. 10.1002/fee.2059

[ece38397-bib-0036] Merriman, R. B. (1962). The rise of the Spanish empire in the old world and in the new. Cooper Square Publishers.

[ece38397-bib-0037] Nash, S. V. (1993). Sold for a song: The trade in Southeast Asian Non‐CITES birds. Traffic International.

[ece38397-bib-0038] Nijman, V. (2010). An overview of international wildlife trade from Southeast Asia. Biodiversity and Conservation, 19(4), 1101–1114. 10.1007/s10531-009-9758-4

[ece38397-bib-0039] Olson, D. M. , Dinerstein, E. , Wikramanayake, E. D. , Burgess, N. D. , Powell, G. V. N. , Underwood, E. C. , D’amico, J. A. , Itoua, I. , Strand, H. E. , Morrison, J. C. , Loucks, C. J. , Allnutt, T. F. , Ricketts, T. H. , Kura, Y. , Lamoreux, J. F. , Wettengel, W. W. , Hedao, P. , & Kassem, K. R. (2001). Terrestrial ecoregions of the world: A new map of life on earth. BioScience, 51(11), 933–938. 10.1641/0006-3568(2001)051[0933:TEOTWA]2.0.CO;2

[ece38397-bib-0040] Orme, C. D. L. , Davies, R. G. , Olson, V. A. , Thomas, G. H. , Ding, T.‐S. , Rasmussen, P. C. , Ridgely, R. S. , Stattersfield, A. J. , Bennett, P. M. , Owens, I. P. F. , Blackburn, T. M. , & Gaston, K. J. (2006). Global patterns of geographic range size in birds. PLoS Biology, 4(7), e208. 10.1371/journal.pbio.0040208 16774453 PMC1479698

[ece38397-bib-0041] R Core Team (2021). R: A language and environment for statistical computing. R Foundation for Statistical Computing. http://www.R‐project.org/

[ece38397-bib-0042] Reino, L. , Figueira, R. , Beja, P. , Araújo, M. B. , Capinha, C. , & Strubbe, D. (2017). Networks of global bird invasion altered by regional trade ban. Science Advances, 3(11), e1700783. 10.1126/sciadv.1700783 29181443 PMC5699901

[ece38397-bib-0043] Ribeiro, J. , Reino, L. , Schindler, S. , Strubbe, D. , Vall‐llosera, M. , Araújo, M. B. , Capinha, C. , Carrete, M. , Mazzoni, S. , Monteiro, M. , Moreira, F. , Rocha, R. , Tella, J. L. , Vaz, A. S. , Vicente, J. , & Nuno, A. (2019). Trends in legal and illegal trade of wild birds: A global assessment based on expert knowledge. Biodiversity and Conservation, 28(12), 3343–3369. 10.1007/s10531-019-01825-5

[ece38397-bib-0044] Romagosa, C. M. , Guyer, C. , & Wooten, M. C. (2009). Contribution of the live‐vertebrate trade toward taxonomic homogenization. Conservation Biology, 23(4), 1001–1007. 10.1111/j.1523-1739.2009.01194.x 19627323

[ece38397-bib-0045] Romero‐Vidal, P. , Hiraldo, F. , Rosseto, F. , Blanco, G. , Carrete, M. , & Tella, J. L. (2020). Opportunistic or non‐random wildlife crime? Attractiveness rather than abundance in the wild leads to selective parrot poaching. Diversity, 12(8), 314. 10.3390/d12080314

[ece38397-bib-0046] Russell‐Wood, A. J. R. , & Russell‐Wood, A. J. R. (1998). The Portuguese empire, 1415–1808: A world on the move. Johns Hopkins University Press.

[ece38397-bib-0047] Seebens, H. , Blackburn, T. M. , Dyer, E. E. , Genovesi, P. , Hulme, P. E. , Jeschke, J. M. , Pagad, S. , Pyšek, P. , Winter, M. , Arianoutsou, M. , Bacher, S. , Blasius, B. , Brundu, G. , Capinha, C. , Celesti‐Grapow, L. , Dawson, W. , Dullinger, S. , Fuentes, N. , Jäger, H. , … Essl, F. (2017). No saturation in the accumulation of alien species worldwide. Nature Communications, 8, 14435. 10.1038/ncomms14435 PMC5316856 28198420

[ece38397-bib-0048] Severinghaus, L. L. , & Chi, L. (1999). Prayer animal release in Taiwan. Biological Conservation, 89(3), 301–304. 10.1016/S0006-3207(98)00155-4

[ece38397-bib-0049] Shepherd, C. R. (2006). The bird trade in Medan, north Sumatra: An overview. BirdingASIA, 5, 16–24.

[ece38397-bib-0050] Shieh, B. , Lin, Y. , Lee, T. , Chang, C. , & Cheng, K. (2006). Pet trade as sources of introduced bird species in Taiwan. Taiwania, 51(2), 81–86.

[ece38397-bib-0051] Signorile, A. L. , Wang, J. , Lurz, P. W. W. , Bertolino, S. , Carbone, C. , & Reuman, D. C. (2014). Do founder size, genetic diversity and structure influence rates of expansion of North American grey squirrels in Europe? Diversity and Distributions, 20(8), 918–930. 10.1111/ddi.12222

[ece38397-bib-0052] Su, S. , Cassey, P. , & Blackburn, T. M. (2014). Patterns of non‐randomness in the composition and characteristics of the Taiwanese bird trade. Biological Invasions, 16, 2563–2575. 10.1007/s10530-014-0686-1

[ece38397-bib-0053] Su, S. , Cassey, P. , & Blackburn, T. M. (2015). The wildlife pet trade as a driver of introduction and establishment in alien birds in Taiwan. Biological Invasions, 18(1), 215–229. 10.1007/s10530-015-1003-3

[ece38397-bib-0054] Su, S. , Cassey, P. , Vall‐llosera, M. , & Blackburn, T. M. (2015). Going cheap: Determinants of bird price in the Taiwanese pet market. PLoS One, 10(5), e0127482. 10.1371/journal.pone.0127482 26017386 PMC4445911

[ece38397-bib-0055] 2011). The Avicultural Society of Australia Inc . Member's notices birds for sale & wanted to buy. April 2011 to November 2013. Australian Aviculture.

[ece38397-bib-0056] The Queensland Finch Society Inc . (2012). Finch price guide 2012–2014. Finch News.

[ece38397-bib-0057] The United Bird Societies of South Australia Inc . (2003). Bird price guide (1st to 7th ed.). The United Bird Societies of South Australia Inc.

[ece38397-bib-0058] Turbé, A. , Strubbe, D. , Mori, E. , Carrete, M. , Chiron, F. , Clergeau, P. , González‐Moreno, P. , Le Louarn, M. , Luna, A. , Menchetti, M. , Nentwig, W. , Pârâu, L. G. , Postigo, J.‐L. , Rabitsch, W. , Senar, J. C. , Tollington, S. , Vanderhoeven, S. , Weiserbs, A. , & Shwartz, A. (2017). Assessing the assessments: Evaluation of four impact assessment protocols for invasive alien species. Diversity and Distributions, 23(3), 297–307. 10.1111/ddi.12528

[ece38397-bib-0059] Turner, G. (2003). British cultural studies: An introduction (3rd ed.). Routledge.

[ece38397-bib-0060] Vall‐llosera, M. , & Cassey, P. (2017). Physical attractiveness, constraints to the trade and handling requirements drive the variation in species availability in the Australian cagebird trade. Ecological Economics, 131, 407–413. 10.1016/j.ecolecon.2016.07.015

[ece38397-bib-0061] Vall‐Llosera, M. , & Su, S. (2018). Trends and characteristics of imports of live CITES‐listed bird species into Japan. Ibis, 161(3), 590–604. 10.1111/ibi.12653

[ece38397-bib-0062] Vall‐llosera, M. , Woolnough, A. P. , Anderson, D. , & Cassey, P. (2017). Improved surveillance for early detection of a potential invasive species: The alien Rose‐ringed parakeet *Psittacula krameri* in Australia. Biological Invasions, 19(4), 1273–1284. 10.1007/s10530-016-1332-x

[ece38397-bib-0063] Westphal, M. I. , Browne, M. , MacKinnon, K. , & Noble, I. (2008). The link between international trade and the global distribution of invasive alien species. Biological Invasions, 10(4), 391–398. 10.1007/s10530-007-9138-5

[ece38397-bib-0064] Wong, J.‐J. , Wu, Y.‐C. , & Tang, P.‐C. (2012). The management and trade status of parrots in Taiwan (pp. 243). Council of Agriculture, Executive Yuan. http://www.coa.gov.tw/view.php?catid=2446191

[ece38397-bib-0065] Wyatt, T. (2009). Exploring the organization of Russia Far East's illegal wildlife trade: Two case studies of the illegal fur and illegal falcon trades. Global Crime, 10(1–2), 144–154. 10.1080/17440570902783947

[ece38397-bib-0066] Xu, W. G. , & Dong, R. M. (2007). The four most popular songbirds in China. ShangHai Scientific and Technological Literature Publishing House Co. Ltd.

[ece38397-bib-0067] Yen, S. (2011). An inventory of the current status of the wildlife importation in Taiwan. Forestry Bureau, Council of Agriculture Executive Yuan.

